# Suitability of botanical extracts as components of complex mixtures used in herbal tea infusions—challenges and opportunities

**DOI:** 10.3389/fphar.2022.1013340

**Published:** 2022-11-07

**Authors:** T. Brendler, J. A. Brinckmann, M. Daoust, H. He, G. Masé, K. Steffan, M. Williams

**Affiliations:** ^1^ Traditional Medicinals Inc., Rohnert Park, CA, United States; ^2^ Department of Botany and Plant Biotechnology, University of Johannesburg, Johannesburg, South Africa

**Keywords:** extract, infusion, hygroscopicity, solubility, dispersibility, distribution, quality control, supply chain

## Abstract

Herbal tea is a mainstay dosage form in practically all systems of traditional medicine and widely used in modern alternative and complementary medicine. Incorporating botanical extracts into herbal tea formulations is of vital interest to manufacturers as it allows for the use of herbal ingredients that would otherwise not be suitable for the dosage form, for instance, dosing requirements, solubility in water, sensory constraints *etc.* Furthermore, reducing the amount of ingredients in a formula increases compliance with dosing recommendations and thus therapeutic benefit. However, formulating with botanical extracts comes with challenges, ranging from sourcing ingredients of appropriate quality, developing suitable methods for quality control with combinations of (herbal) ingredients, processing constraints such as hygroscopicity, solubility, dispersibility, homogeneity of distribution, and packaging machinability, all the way to stability required for hot-water infusion. We report on experiences with overcoming such challenges in a set of examples and provide guidance to the extract industry on how to tap into the bagged tea sector with better suited or tailor-made solutions for the formulator.

## Introduction

Herbal tea, prepared by decoction, infusion, or maceration, is a dosage form widely used in different systems of traditional medicine since ancient times. This is especially well documented in Traditional Chinese Medicine (TCM) (Chen, 2002; Lu, 2002; Pan et al., 2022). Traditional herbal tea infusions were originally prepared using bamboo infusers (Dubrin, 2010). Innovations led to infusers or strainers made of different metals (Antol, 1995). An early 20th century innovation was the filter tea bag (Bajaj, 2016) followed in 1929 with the invention of the first tea packing machine by Adolf Rambold for the German company Teekanne (Block et al., 2016). For much of the 20th century, herbal medicinal teas in American and European pharmacies were either single-herb teas or traditional formulations, composed of several dried and cut herbs.

In the latter part of the century, some companies began to innovate by admixing other types of substances into complex mixtures of herbs prepared in tea-bag-cut (TBC) particle size. These other substances included, for example, aroma, color, and flavor agents such as mixtures of essential oils carried by a range of functional excipients, and eventually dry extracts of herbs and dry plant juices. The secondary processing of herbs of different types and plant parts (flowering aerial parts, fruit, inflorescence, inner tree bark, leaf, root, and rhizome) into suitable free-flowing particles of uniform length, size, and density that function as components of a homogeneous mixture, from the blender to the hopper and from packaging machine into each individual filter tea bag, is an art and a science. The mixing of dry extracts with tea-bag-cut herbs presents a range of physical (e.g., hygroscopicity) and rheological (e.g., flow rate) challenges during manufacturing, as well as effects on infusion kinetics, dissolution, and dispersion of extractive matter through the tea bag filter paper, uniformity of mass, and analytical challenges, among others.

In the early 1990s, the herbal tea company Traditional Medicinals began to innovate and experiment with herbal tea formulations, that were composed of dried tea-bag-cut botanicals in combination with various types of granulated dry extracts, essential oils carried on starch and resin/botanical gum, dried fruits, and dry fruit juice granules. This article outlines some of the lessons learned over 3 decades of practical experience in the formulation of complex herbal medicinal tea infusion products, when combining botanical substances of varying physical properties and performance characteristics.

Meadowsweet (*Filipendula ulmaria* L.), echinacea (*Echinacea purpurea* (L.) Moench.), the TCM formula Biyan Pian, ashwagandha (*Withania somnifera* (L.) Dunal), licorice (*Glycyrrhiza* spp.), elderberry (*Sambucus nigra* L.), eleuthero (*Eleutherococcus senticosus* (Rupr. and Maxim.) Maxim.), hemp (*Cannabis sativa* L.) and reishi (*Ganoderma lucidum* Karst) extracts will exemplify specific extract characteristics in the following.

## Formulation

Traditional principles of formulation for herbal infusions emphasize clinical efficacy, combining the primary and secondary actions of their components to achieve desired therapeutic outcomes (Hoffmann, 2003). Single herbs with their diverse pharmacological components can provide a balanced complement of actions for a given therapeutic outcome: for example, meadowsweet herb yields key active constituents, including salicylates and quercetin-derived polyphenolic glycosides, into a simple aqueous infusion at 90–100°C steeped for 10–15 min (Harbourne et al., 2009). As a result, a teabag infusion made from 2 g of comminuted meadowsweet herb per day can be expected to be effective, as noted by the European Medicines Agency (EMA) in its therapeutic labeling monograph (European Medicines Agency, 2011). For reference, typical total teabag weights in commerce range from 1.5–2.0 g.

This, however, may not be the case for more complex mixtures or for ingredients that are not traditionally suitable for simple infusion as above. Although the amounts can vary depending on the specifics of a formulation, the doses required to achieve therapeutic efficacy for polyherbal formulations can in some cases exceed those that a teabag infusion product can deliver, requiring an impractical number of teabags to be consumed on a daily basis. Even single herbal ingredients, such as echinacea above-ground parts, may require a dose too high to be practical in a teabag indicated for infusion. Additionally, certain ingredients, such as medicinal mushrooms, or certain plant parts, such as roots or barks, present a challenge to infusion and are traditionally prepared by decoction (long-term simmering (Green, 2011). This makes them poor candidates for inclusion in an herbal tea infusion, which is typically steeped in hot water for about 10 min and not simmered. Finally, crucial components of an herbal ingredient’s active chemistry (such as the cannabinoids from hemp) may be poorly soluble in water at any temperature, whether simmered or simply infused.

Extracts of medicinal herbs and mushrooms can serve as a viable option for addressing these challenges when formulating for delivery in double-pouch filter teabags. Extracts may be used as active components of the formulation, or as flavor corrigents to modify the final sensory experience of the infusion. The authors have found that the following are important considerations when formulating teabag infusion products with extracts:

- The types and characteristics of the extract, including extraction process and solvents used,
- Normalization and/or standardization of the extract, including use of emulsifiers, and
- Preparing the extract for use in double-pouch teabag format, including relevant parameters that minimize complications when manufacturing at scale.

### Types and characteristics of suitable extracts

Extracts can be obtained using a variety of techniques, solvents, and extraction parameters. At its most basic, extraction involves exposing comminuted or powdered plant material to a solvent, under variable conditions of time, temperature, and pressure depending on the extraction system. Solvents used can be highly variable, with their unifying characteristic being an ability to penetrate fibrous tissues and cell walls to allow constituents to dissolve down their concentration gradients into a homogeneous, equilibrated solution (List and Schmidt, 1989). After a defined period, the solution is separated from the herbal material through centrifugation, filtration, and/or pressing, yielding a liquid extract. This extract is concentrated, through evaporation of the solvent, to a thick, soft substance (*extractum spissum*), the native extract, which can be further dried into a powder (*extractum siccum*). The ratio of the mass of initial material to the mass of native extract is known as the drug extract ratio (DER), and is roughly indicative of the degree of concentration of the extract (List and Schmidt, 1989).

The solvents used for extraction vary widely. The authors work with extracts produced under the guidance of the National Organic Program (NOP) of the United States, which allows water, ethanol, and carbon dioxide as acceptable solvents/solvent systems (United States Department of Agriculture, 2022). The choices of solvent, extraction time, and pressure, are determined in large part by the traditional use record and modern insight into the polarity of the chemical constituents of interest for the ingredient(s) being extracted (Abubakar and Haque, 2020). On the polar side of the spectrum, as noted in the example of meadowsweet above, water is adequate as a solvent: polar constituents are extracted well, and extraction can be further enhanced by increasing temperature, pressure, and/or agitation. On the non-polar side of the spectrum, ethanol (95% m/m) and carbon dioxide maximize the extraction of oil-soluble non-polar constituents due to the lipophilic nature of the solvents. Mixtures of water and ethanol (hydroalcoholic solvents) allow for extraction that favors a range of chemical constituents with differing polarities (both polar and slightly non-polar). Carbon dioxide, under different conditions of temperature and pressure, can yield different non-polar fractions from a given ingredient. A notable example is hemp, using supercritical carbon dioxide at varying conditions of temperature, pressure, and solvent flow rate, extracts with high cannabinoid or, conversely, high terpene content can be obtained (Baldino et al., 2020). For the purposes of formulation, matching the polarity of the desired chemistry of the extract with the polarity and extraction parameters of the solvent yields an extract that best matches the desired phytochemical profile.

On the one hand, by choosing specific solvents and/or temperature and pressure parameters, a specific subset of chemistry can be favored and concentrated during the extraction process. This can be useful in the production of standardized extracts which require a consistent, quantified amount of certain marker compounds. Using pharmacopoeial and/or proprietary methods, these compounds can then be assayed to ensure the extract meets the requisite strength specifications. On the other hand, when producing traditional native extracts (100% extractive matter), the aim is to recover a content and composition profile that matches as close as possible to that of the starting raw material with regards to active constituents (List and Schmidt, 1989). This can be seen in the production of TCM aqueous dry extracts. After extraction, the *extractum siccum* is compressed into granules with a defined DER range. This enables practitioners to make a dosage correlation between the traditional preparation (decoction of the raw ingredients) and the modernized extract granules.

For example, a TCM formulation known as Biyan Pian [consisting of xanthium fruit (*Xanthium sibiricum* Patrin ex Widder); magnolia flower (*Magnolia biondii* Pamp., *Magnolia denudata* Desr., or *Magnolia sprengeri* Pamp.); siler root (*Saposhnikovia divaricata* (Turcz.) Schischk.); forsythia fruit (*Forsythia suspensa* Vahl.); chrysanthemum flower (*Chrysanthemum indicum* L.; Schisandra fruit *Schisandra chinensis* (Turcz.) Baill; Platycodon root *Platycodon grandiflorus* A. DC.); fragrant angelica root (*Angelica dahurica* (Fisch. ex Hoffm.) Benth. and Hook.); anemarrhena rhizome (*Anemarrhena asphodeloides* Bunge); schizonepeta herb (*Nepeta tenuifolia* Benth.; and Chinese licorice root (*Glycyrrhiza uralensis* Fisch. ex DC.) (Chinese Pharmacopoeia Commission, 1997) is indicated for rhinosinusitis and respiratory congestion. It is traditionally prepared by combining the herbal ingredients and decocting, or simmering, the mixture for a prolonged period, making it impractical for use in teabag infusion products both because of its preparation and the mass of herbal ingredients required. A dry aqueous extract (using water as the only solvent) of Biyan Pian with an average DER of 8:1 addresses both these concerns by pre-extracting the herbal ingredients so that prolonged simmering is no longer required, and by concentrating the extract roughly eight-fold through evaporation of the water solvent. In this way, by using the extract in a teabag, approximately 8-times the amount of herb equivalents is delivered into the aqueous infusion when the teabag is steeped for 10–15 min at 90–100°C.

While the example above uses water as the only extraction solvent, other choices are available for other herbal ingredients. For example, a dry extract prepared from the root of echinacea uses a hydroethanolic solvent (30%–40% m/m certified organic ethanol). This solvent is selected for two reasons: first, historical evidence emphasizes the use of the hydroalcoholic tincture as an effective medicinal preparation of this herb (King and Felter, 1898). Second, modern analytical chemistry emphasizes the non-polar phenolic and alkamide fractions of the root (Spelman et al., 2009). After extraction in the hydroethanolic solvent, a dry extract is produced, which is incorporated into teabag infusion products designed to stimulate immunologic function (European Medicines Agency, 2015). In this case, the use of the extract allows for delivery of an efficacious amount of constituents (among them, phenolic compounds and alkamides) from fibrous roots in a non-traditional format.

In certain cases, an ingredient will have a traditional use record and/or modern pharmacognostic evidence pointing to poor water solubility/dispersibility. For example, the root of ashwagandha is traditionally ingested as a powder (i.e., not infused or decocted), or if prepared by simmering, a lipid-rich solvent such as milk or ghee (clarified butter) is used alongside water (Pole, 2006). While aqueous extraction at high temperature and pressure may yield an extract rich in compounds considered medicinally relevant (Hooda et al., 2022), it is not guaranteed that the dry extract and its phytochemistry will adequately disperse into an infusion. In another example, the extract of hemp is produced using solvents such as supercritical carbon dioxide or 95% ethanol (m/m) to maximize the yield of non-polar cannabinoids, yielding a soft native extract (*extractum spissum*) with a variable DER. However, this extract is wholly insoluble in water, and does not disperse through a teabag. Through the use of post-extraction processing techniques, including blending with excipients and emulsification of the native extracts, these dispersibility/solubility challenges can be overcome, allowing for the use of a range of different extracts in teabag infusion products.

### Normalization, standardization, and post-extraction processing of native extracts

Due to natural variability of the herbal ingredients used to manufacture extracts (Pant et al., 2021), and also due to the desire for a consistent, quantifiable amount of active compounds, a native extract may undergo a process of normalization and/or standardization. This can be accomplished by blending the native extract with an excipient such as maltodextrin. In this case, the excipient allows the composition of the final extract to be adjusted to a normalized DER and/or a standardized content of active compounds. For example, the hydroalcoholic extract of echinacea root mentioned above can be normalized so that the final internal composition is between 90%–100% native extract, and 0%–10% excipient. This allows for a consistent level of phenolic compounds and alkamides, which are assayed for compliance with internal specifications (based on pharmacopoeial standards) before the extract is used to manufacture finished products. A normalized extract, which declares the native extract DER range, the excipient content range, and potentially the final normalized DER, can be used to assess a final extract’s phytoequivalence for the purpose of assessing therapeutic efficacy either alone or as part of a formulation (Monagas et al., 2022).

Beyond the process of normalization and standardization described above, a native extract can undergo additional processing after extraction. This is most relevant for extracts prepared using ethanol or supercritical carbon dioxide as solvents but can be explored for any extract that shows poor solubility/dispersibility in the teabag infusion product format. Emulsification of these native extracts significantly improves their performance in an infusion and can be achieved by micro-encapsulating the soft extract into micelles. Previous research has identified phospholipids, such as those obtained from sunflower (*Helianthus annuus* L.) (Komaiko et al., 2015), saponins, such as those obtained from the soapbark tree (*Quillaja saponaria* Molina) (Schreiner et al., 2021), and other emulsifiers as promising candidates for the creation of spontaneous micro-emulsions for oil-in-water systems. The types and characteristics of suitable extracts, including the use of different excipients for different types of extracts and applications, are summarized in Table 1.

**TABLE 1:** Types and characteristics of extracts suitable for use in teabag infusion products.

Polarity of active compounds	Solvent System	Qualities/composition	Native extract	Normalized/Standardized extract	Teabag-ready extract
Polar	Aqueous	Excipients	none	maltodextrin, gum acacia	maltodextrin, gum acacia, sugar
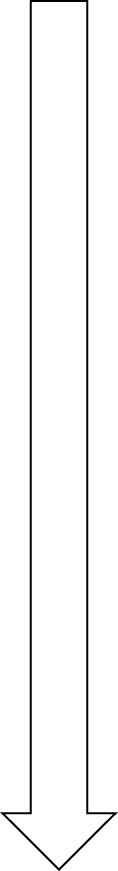		Format	dry powder	dry powder	dry granules
		Infusibility	moderate to high	moderate to high	high
	Hydro-alcoholic	Excipients	none	maltodextrin, gum acacia	gum acacia, lecithin
		Format	dry powder	dry powder	dry granules or flakes
		Infusibility	moderate	moderate to high	high
	Ethanolic	Excipients	none	maltodextrin, gum acacia	gum acacia, lecithin, saponins
		Format	dry powder or soft mass	dry powder or soft mass	dry flakes
		Infusibility	low to moderate	moderate	high
	C02	Excipients	none	lipid carrier	gum acacia, lecithin, saponins
		Format	soft mass or liquid	liquid	dry flakes
Non-Polar		Infusibility	none to low	none to low	moderate to high

Native extracts often present a range of DERs due to natural variability of the starting material. Depending in part on the specific herb to be extracted and its content of water- or ethanol-soluble extractives, these can range from averages of 4–10:1, up to 33:1 (Monagas et al., 2022). Normalized extracts offer a consistent DER by adding variable amounts of excipients. Standardized extracts offer a consistent, quantified amount of marker compound(s). Teabag-ready extracts, which may be normalized or standardized before processing, blend the extract with excipients and emulsifiers to aid in their infusibility. Infusibility ratings are based on the authors’ experience comparing assayed marker compounds in the extract(s) to assayed marker compounds in the infusion.

### Preparing extracts for use in teabag infusion products

Dried or concentrated liquid extracts require preparation before they can be incorporated into blends destined for use in teabag infusion products. Dried extracts (*extractum siccum*) have very small particle sizes which are defined as microfine powders (Pharmaceutical Guidelines, 2022). Size spectra are around 1–3 µm by number though they can range widely from 1–800 µm depending on types of extracts and drying processes (Ulyantsev et al., 2011). On the other hand, herbs used for bagged teas are bulk solids with particle sizes ranging from a few millimeters to a fraction of a millimeter, typically between US. mesh #10 (2 mm) and US. mesh #60 (0.25 mm). Mixing microfine extract powder with TBC herbs widens the particle size distribution of the blend, which can impact blend homogeneity.

Particle size influences the flowability of material. Flowability is the ratio between the cohesive forces making particles stick together and the normal forces breaking them apart (Lawrence, 2017). Coarse particles have a better flowability than finer particles. Blending microfine extract powders with TBC herbs also increases the differences in flowability between the components, which leads to higher segregation of a mixture (Savage and Lun, 1988). Furthermore, microfine powder tends to stick on machine surfaces, which increases the friction between the dosing parts of a tea packaging machine, generates heat, and can seize the packaging machine. Consequently, the efficiency of tea packaging operations decreases.

Therefore, one must take measures to agglomerate powder extract into machinable particle size to mitigate undesirable physical properties and yield a homogeneous mixture with good machinability.

### Extract agglomeration

**FIGURE 1:**
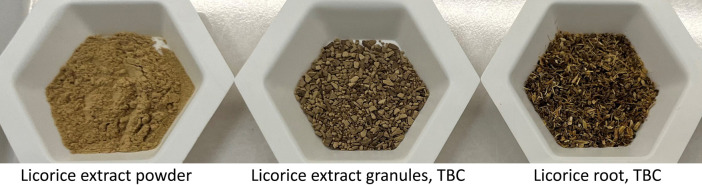
Samples of licorice extract powder, licorice root extract granules and licorice root TBC.

There are multiple technologies for converting a powder extract into a TBC particle size depending on the extract in question. For an aqueous extract, dry granulation by compression/compaction is most often used in the industry, being economical and suitable for heat-sensitive botanical compounds (Sharma, 2017). Dry granulation utilizes two rollers that revolve towards each other to form solid compacts or sheets by hydraulic pressure which are then milled and granulated to TBC particles. Figure 1 shows samples of licorice root aqueous extract powder, licorice root aqueous extract granules, and licorice root TBC to illustrate how licorice root aqueous extract granules have a similar particle size to TBC licorice roots.

The hardness of granules is proportional to compression force. Appropriate granule hardness is critical. The granulation can be processed with excipients (such as maltodextrin, sugars, gum acacia, and starch) for standardization, or to mitigate the separation of the ingredients in a solid mixture during blending and material handling and improve the flow of the mixture in the tea packaging machine. When granules are too fragile/friable, they may break down to powders negatively affecting machinability. When granules are too hard, they may not disintegrate and dissolve sufficiently for full release during the allotted brewing time (Lira Soares et al., 2005).

For an extract prepared by hydroalcoholic and/or supercritical CO_2_ extraction, water solubility is limited though it varies. It is vital to increase the particle size, as well as the solubility/dispersibility, to make it suitable for bagged tea application. A widely used practice is to agglomerate such an extract with appropriate excipients with emulsifying properties and homogenize the slurry before drying and milling to TBC size. Desai et al. reviewed various techniques for producing dry emulsions (Desai and Jin Park, 2005). Depending on the polarity of extract active components, different excipients can be used. The lower the polarity of an extract’s phytochemical constituents, the more emulsification function a carrier needs to provide. For an extract with constituents of very low polarity, emulsifiers are often used to make it dispersible in water. For example, supercritical CO_2_ hemp extract is hydrophobic and dissolves in fat, not in water. It needs the help of excipients with strong emulsifying properties, such as sunflower lecithin, to become dispersible in water. Another example is vanilla pod (*Vanilla planifolia* Andrews) hydroalcoholic extract, which has a lipid-rich nature. It has a thick and pasty consistency: gum acacia and/or maltodextrin can make it dispersible in tea infusion (Havkin-Frenkel and Belanger, 2018).

Concentrated liquid extracts (*extracta spissa*) can sometimes be sprayed onto plant parts (TBC) prior to drying to become incorporated with the plant parts. For example, an elderberry juice extract (*Sambucus nigra* L.) can be applied to echinacea aerial parts (TBC) before drying, which normalizes particle size and mitigates the hygroscopicity of the extract.

Finally, when an herbal mixture contains a very low percentage of aqueous extract, a dry extract can be directly sprayed onto herbal leafy materials (TBC) while mixing. The mechanism consists of coating extract microfine particles onto the surface of comparatively large herbal leaflets. Since the attractive force between two microfine particles in contact is greater than the weight of a microfine particle, the particles stick together on the TBC leaflet, and segregation will not occur (Rhodes, 1990). If necessary, adding a small amount of liquid while spraying helps achieve a better mixing quality.

### Extract granule hygroscopicity

**FIGURE 2:**
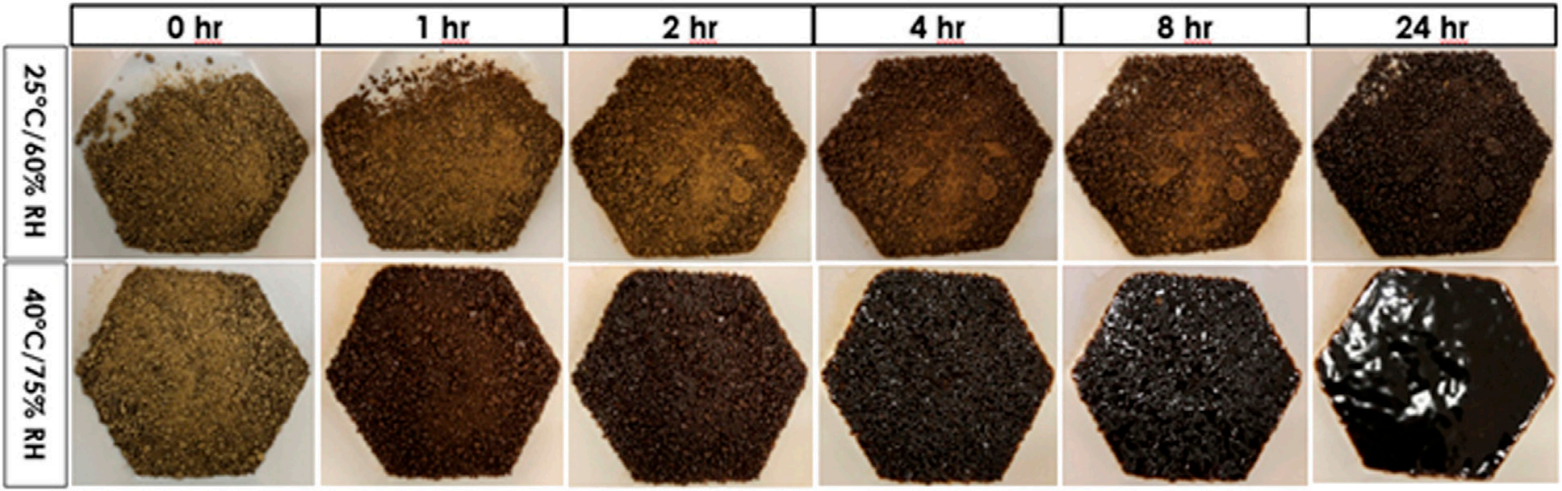
Changes of eleuthero aqueous extract granules when exposed to high temperature and high humidity over time.

Agglomeration helps reduce the surface areas of microfine extract particles and improves its hygroscopicity (Gallo et al., 2015). However, agglomerated extract granules for tea infusion can readily absorb water, form clumps, and become sticky over time when exposed to a moist and warm environment. For example, Eleuthero root aqueous extract granules are free-flowing material. When exposed to 25°C/60% RH, the granules darkened slightly and were free flowing after 1 h, darkened further and started clumping after 2 h, became slightly sticky and continued clumping after 8 h, exhibited further stickiness and started to melt after 24 h. When exposed to 40°C/60% RH, the granules darkened significantly and clumped together after only 1 h, darkened further and became rock hard after 2 h, started to liquefy and became very sticky after 4 h, and completely melted and became extremely sticky after 24 h. Figure 2 depicts the changes of eleuthero aqueous extract granules when exposed to 25°C/60% RH and 40°C/75% RH conditions over 24 h. Clearly, temperature and humidity accelerate the process, and the higher the temperature, the higher the relative humidity, the less desirable the granules become.

The hygroscopic nature of an extract can have negative consequences during material storage, processing, and/or finished product shelf life. In a humid and warm environment, a hygroscopic extract can absorb water during both storage and processing, raising its moisture content enough to make it slightly sticky and more likely to adhere to the components of the machinery (to dose the blend into tea bags), which hampers performance and causes production loss. One must take adequate precautions when weighing and handling extract materials. Once open, the package containing unused extract material must be sealed well and quickly and stored in a cool environment.

Furthermore, the hygroscopic nature of an extract also impacts the quality of the finished product during shelf life. It is critical that the packaging substrate have appropriate barrier properties to prevent water and/or oxygen from penetrating these barriers over time. Therefore, it is essential to package herbal teas containing extracts in a substrate with excellent moisture and oxygen barriers and store them in a cool dry environment to maintain the good organoleptic quality of finished products.

### Extract solubility and dispersibility

**FIGURE 3:**
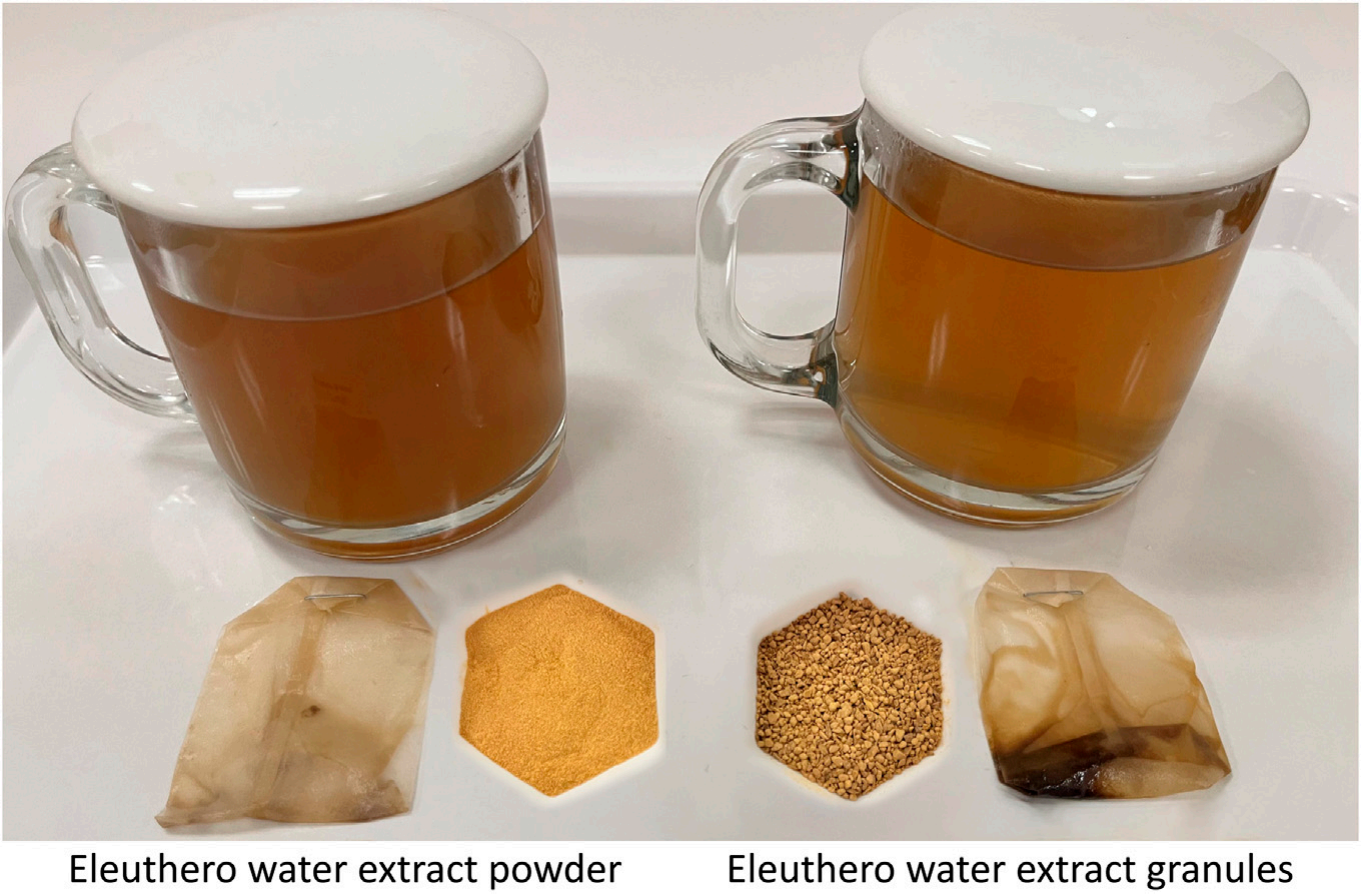
Changes in dispersibility of an extract after granulation.

The solubility of active constituents in an infusion is vital to deliver efficacious products for consumers to experience therapeutic benefits. Theoretically, granules made from an aqueous water extract should be water soluble, but the authors found this is not always the case. Granules often require more time and effort to get solubilized than powder as they must go through a process of disintegration and dissolution (Farakte et al., 2016). Disintegration is achieved by the penetration of water into the compacted powder, disrupting the particle-particle bonds which maintain the structural integrity of granules. For example, eleuthero aqueous extract once compacted to granular form showed decreased solubility in water, as demonstrated in Figure 3. The infusion prepared by steeping the granules in a teabag in freshly boiled water was lighter than was seen with the powder, and the teabag of the granules retained more extract than that of the powder. Any amount of extract left in the teabag directly reduces the efficacy of a product. Therefore, it is sensible to control the granulation processes to optimize the particle size and hardness of granules. This can help ensure final product efficacy, beyond supporting the homogeneity and machinability of herbal blends.

For hydroalcoholic or CO_2_ extracts agglomerated with emulsifiers, active constituents must be dispersible for bioavailability regardless of their low polarity. This requires the use of emulsifiers to create oil-in-water emulsions and stabilize the suspension of small droplets to prevent them from coalescing or coming together to grow larger droplets. Emulsifiers form physical barriers to prevent droplets from coming together. Such emulsions can easily be dispersed in water, though the resultant tea infusions may look cloudy.

**FIGURE 4:**
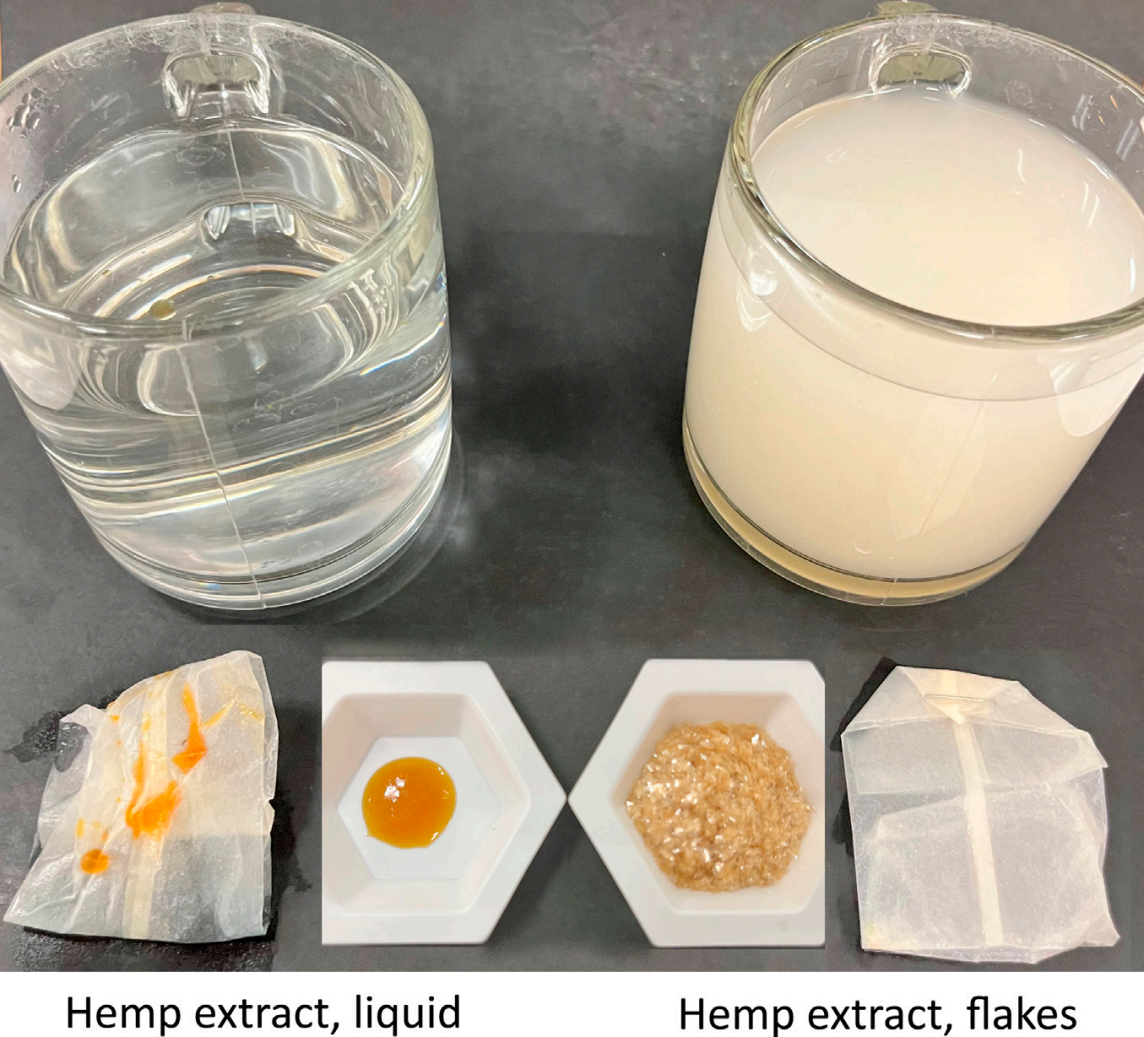
Impact of emulsification on hemp extract dispersibility in water.

A good example is to apply oil-in-water emulsions to hemp extract for tea infusions. Hemp extract is totally insoluble in water, and sunflower lecithin, a phospholipid molecule, is an effective and popular food emulsifier. By agglomerating hemp extract with emulsifiers such as lecithin, gums and other saponin-containing plant materials, an oil-in-water emulsion can be created, which makes hemp extract disperse across the fiber of the teabag and into an aqueous infusion. Figure 4 depicts the power of this emulsification technology on converting oil-soluble material to water-dispersible emulsions. As shown in the pictures on the left, when hemp extract, extracted by supercritical carbon dioxide, was steeped in a teabag in freshly boiled water for 10 min, a few drops of the extract floated on the top of water, and most remained in the teabag. Conversely, the pictures on the right show that, when the emulsified hemp extract flakes went through the same steeping process, essentially all the hemp extract was dispersed into the water with little left in the teabag. The formation of oil-in-water emulsions enabled cannabinoids to disperse into water, increasing the bioavailability of the hemp extract.

## Quality assurance and control

Quality testing of botanical extracts, the raw materials from which they are derived, and finished products into which they are formulated is paramount to establishing safety and efficacy. Quality testing requirements, governed by national and international regulations, evaluate identity, purity, strength, composition, and limits on contaminants. The establishment of pharmacopoeial-based specifications is an effective strategy for the assurance of medicinal grade ingredient quality.

### Identity testing of extracts in infusion formulas

Confirmation of identity is critical to botanical quality testing, as is widely reflected in regulations governing the manufacture and marketing of dietary supplements and herbal medicinal products (European Commission, 2003; United States Food and Drug Administration, 2022). The identity methods most cited within pharmacopoeias are of two types: macro/microscopic analysis and High Performance Thin Layer Chromatography (HPTLC) (Noviana et al., 2022). While diagnostic anatomical and cellular features needed for macro/microscopic identification are not maintained through the process of extraction, a botanical extract’s chemical fingerprint acts as a link to the identity of the raw material. HPTLC allows for comparative visualization of multiple chemical fingerprints (chromatograms) across a single plate. The opportunity for inclusion of a variety of sample types along with botanical and chemical references, makes it the analytical method of choice for establishing identity of botanical extracts.

**FIGURE 5:**
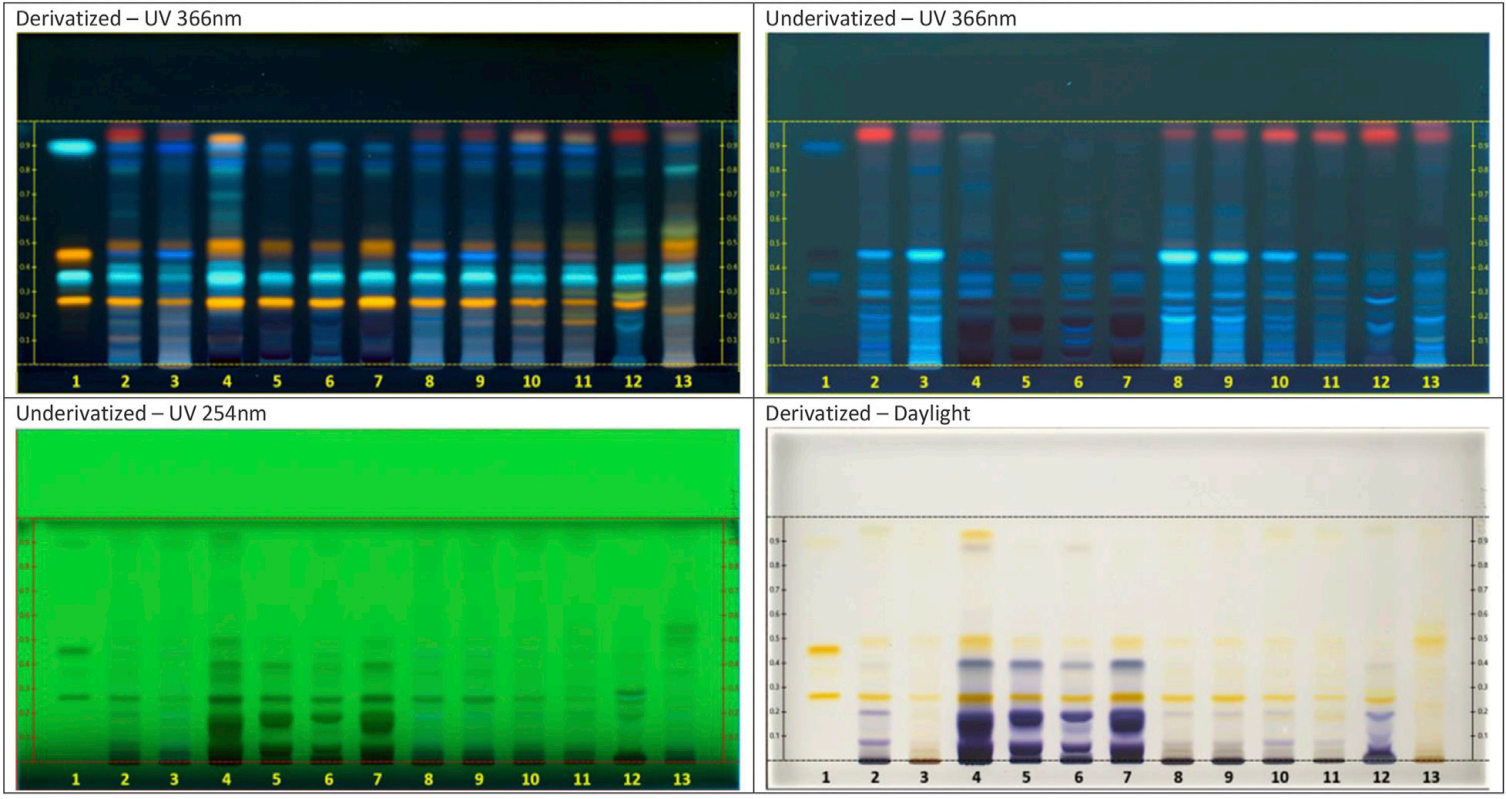
Chromatograms for the identification of elderberry. Chemical reference standards: (1) caffeic acid, hyperoside, chlorogenic acid, rutin; botanical reference materials (BRM): (2) *Sambucus* nigra AHP (whole fruit); (3) *S. nigra* Chromadex (ground fruit); (4) *S. nigra* Dry Extract USP (alcohol extract); commercial samples: (5) elderberry extract (lot 15700004); (6) elderberry juice powder (lot 0609-17528); (7) elderberry juice liquid (lot 202008268001); (8) elderberries, TBC (lot 0609-16944-288439-01); (9) elderberries, ground (lot 0609-17363-295537-01); (10) elderberries, whole (lot 2038917); (11) elderberries, TBC (lot 2038920); BRM (12) S. cerulea AHP (whole fruit); (13) S. cerulea Chromadex (ground fruit).

Figure 5 shows HPTLC plate images of the United States Pharmacopoeia (USP) monograph method for the identification of elderberry dry extract by HPTLC (United States Pharmacopoeia Convention, 2019). Visualized at different wavelengths prior to and following derivatization, the plate compares a variety of elderberry extract sample types: alcoholic, juice concentrate (liquid vs powder) relative to raw fruit at various levels of processing (whole, TBC, and ground). Chemical and authenticated botanical reference materials were included for the purpose of establishing identity, along with references for a distinguishable alternate species.

The comparison of extract samples to references by HPTLC demonstrates consistency of the chemical fingerprint across extract preparations as well as the presence of marker compounds such as rutin and chlorogenic acid (see Figure 5). HPTLC can be a powerful tool for distinguishing alternate species or plant parts as potential adulterants, in addition to detection of other sources of adulteration of known or unknown origin. For example, with increased pandemic-related demand for elderberry products, there is growing concern over economically motivated adulteration with black rice (*Oryza sativa* L.) extract (Gafner et al., 2021).

Availability of extract-specific methods in pharmacopoeias can present a potential challenge, as botanical monographs are often focused on the raw botanical. While extract-specific monographs are commonly provided within the USP and becoming increasingly available from pharmacopoeias, issues related to incongruency of the extract type may still arise. As previously discussed, aqueous extracts are common herbal tea ingredients due to their water-soluble properties. Botanical extract monographs; however, are often developed based on alcoholic or hydroalcoholic extracts. We find that monograph methods developed for a raw botanical or alternate extract type can often be adapted for use with an aqueous extract. The inclusion of proper samples aids in confirming the method as fit for purpose.

**FIGURE 6:**
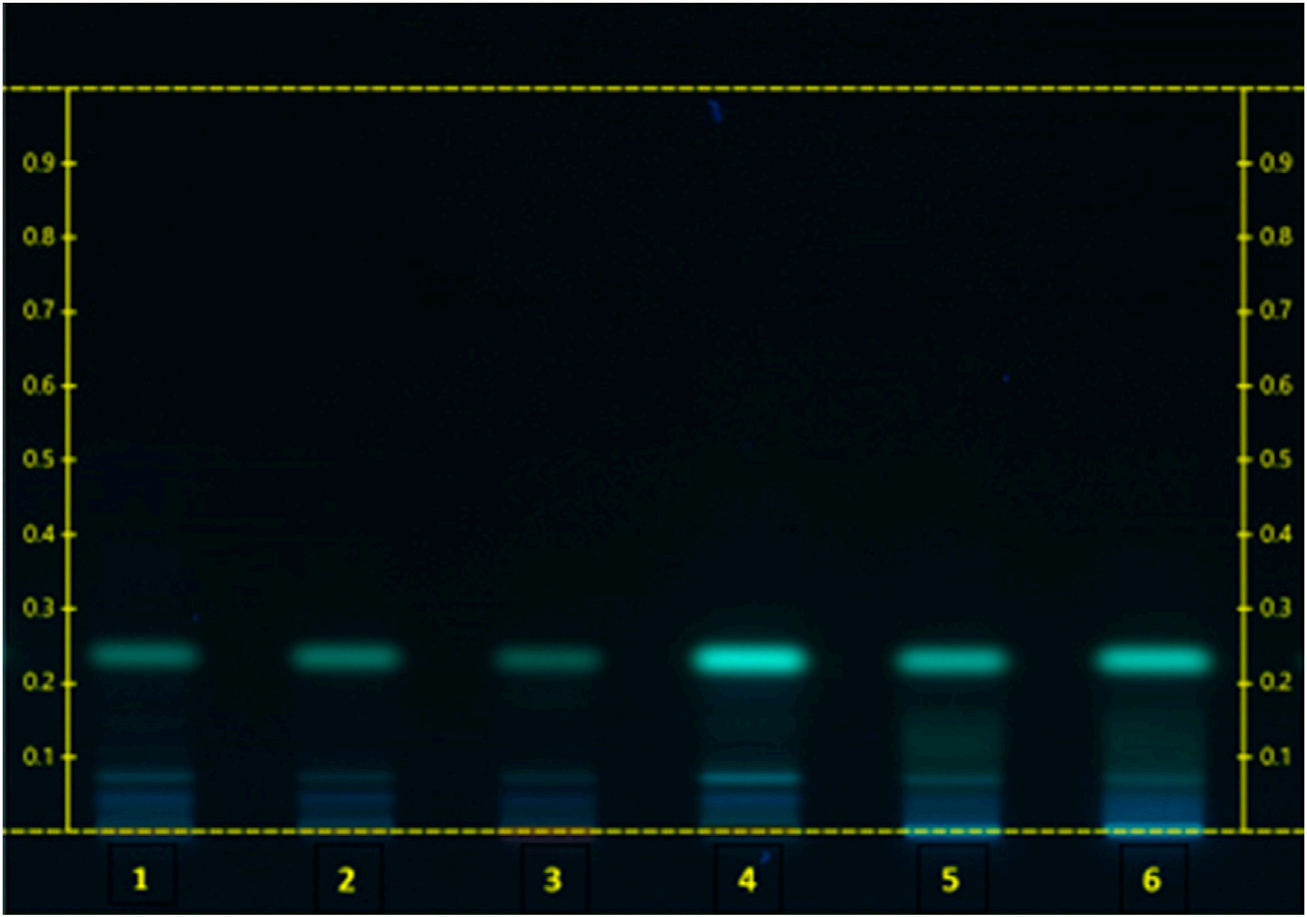
Chromatogram reishi. PPRC 2015 HPTLC ID Method 2: BRM: (1) G. lucidum AHP (whole fruiting body); (2) G. lucidum fruiting body Dry Extract USP; Samples: (3) Reishi whole raw material; (4) Reishi powdered raw material; (5) Reishi aqueous extract powder; (6) Reishi aqueous extract granules.

Extract suppliers able to provide retain samples of their raw materials are of value to the herbal tea manufacturer for the purpose of method adaptation as well as traceability throughout processing. For example, during the ingredient substantiation phase of a granulated aqueous dry extract of reishi mushroom fruiting body for which no specifically applicable extract monograph was available, the supplier provided representative raw whole material (suitable for orthogonal testing by macro/microscopy) along with material sampled at sequential stages of processing: raw powdered fruiting body, powdered extract, and granulated extract. HPTLC analysis of these samples combined with authenticated references confirmed continuity of identity throughout processing while also aiding in pharmacopoeial method evaluation in a case where extract-specific monographs were unavailable (Figure 6).

In addition to identity testing of single ingredient extracts, HPTLC is a valued technique for determination of extract identity when combined with other herbal ingredients (Reich and Frommenwiler, 2021; Frommenwiler et al., 2022; Noviana et al., 2022). An elderberry juice extract applied to a substrate of echinacea aerial parts to regulate particle size and mitigate hygroscopicity requires an identification method capable of distinguishing both herbal components of the combined ingredient. HPTLC analysis of the combined ingredient compared with the individual components accomplishes this by determining the chromatographic contribution of each component to the combined chemical fingerprint and determines diagnostic bands for each component. The same principle is possible for detection of extract ingredients within a more complex multi-herb tea blend; however, the possibility of matrix interference increases with blend complexity.

In evaluation of finished product composition, challenges with matrix interference and the detection of herbal extract ingredients may arise. As described in reference to detection of adulteration by HPTLC, ability to detect declines as levels within a mixture drop below 5% (Ankli et al., 2008). Limits of detection by HPTLC may contribute to identity challenges as well. Due to machinability constraints and concentrated nature, extracts are often formulated in blends at low levels. Our experience has also shown challenges with detection by HPTLC are more likely to occur when extract ingredients are formulated at less than 5% of a blend’s total composition.

In addition, when assessing identity in a finished blend, we have experienced HPTLC limitations when trying to qualitatively distinguish a raw botanical and an extract of the same botanical (Reich and Frommenwiler, 2021). A solution we have employed uses a quantitative technique such as HPLC and a marker compound. The pharmacopoeial strength marker compound glycyrrhizic acid in licorice root was quantified by HPLC in a multi-herbal tea blend containing both raw licorice root and a licorice root dry extract standardized for glycyrrhizic acid content. In this case, HPTLC would not distinguish the two ingredients in a chromatogram generated on the blend. Assaying for glycyrrhizic acid content in the finished blend was performed as indirect confirmation of the extract ingredient based on overall content level.

## Supply chain development for special extracts

It has been the experience of the authors of this paper that the procurement of an extract, one that is suitable for use as one component of a medicinal herbal tea formulation (in combination with dried herbs), generally requires the development of a special extract. The standard offerings of extraction houses are produced with an aim towards usage in solid dosage forms, i.e., capsules, lozenges, pastilles, pills, and tablets.

While extraction houses servicing the phytopharmaceutical sector may produce extracts using pharmacopeial quality starting materials under pharmaceutical drug GMP conditions, that may also meet quality and ingredient performance requirements, such companies are generally less experienced in the production of extracts that conform to the requirements of sustainability standards, such as use of purified water and certified organic ethanol as extraction solvents, and, if needed, certified organic and non-GMO verified excipient materials. On the other hand, extraction houses that target the sustainable products sector (e.g., non-GMO, organic and fair trade) are generally servicing customers that do not require pharmacopeial quality ingredients and/or controlled content or minimum-maximum range of active principles. The target customers for organic and fairtrade herbal extracts are generally natural cosmetic and natural food product companies, and not necessarily herbal medicinal product companies.

We have found that upfront technical cooperation between the Quality and R&D personnel of both the extraction house and the herbal tea brand is necessary in many cases in order to co-develop a special extract fit-for-purpose. We have also found that Sourcing personnel at the herbal tea brand may need to become involved and coordinate with the Procurement personnel at the extraction house. That is because, in some cases, the commercial supply of herbal starting material is for the most part produced and traded in the conventional market, without sustainability documentation (such as fairtrade, non-GMO, and organic).

While an extraction house may be willing to produce a special extract according to a brand’s specification requirements for content, composition, quality, strength, supply chain visibility and sustainability, so long as the estimated annual demand volume is sufficiently high enough to warrant the development of a special extract, the commercial availability of certified organic herbal raw materials may be insufficient. In such cases, sustainable supply chain development work becomes necessary.

Once the estimated annual demand of the special extract and the theoretical native DER range are known, the annual requirement for starting materials may be calculated. Sourcing personnel will first determine if any producer operations (both farming and wild collection operations) are known to reliably produce pharmacopoeial quality herbal drug materials (of the required species and plant parts), what the operation’s current capacity is, whether they have an ability to increase farm hectarage or controlled wild collection area, a time frame for scaling up, and what, if any guarantees must be made in advance in order to cause a producer to scale up production of a particular quality of herb.

Secondly, if the producer operation is not in the certified sustainable ingredient sector, financial and technical support to assist the producer with successful implementation of certain voluntary sustainability standards towards audit and certification may be necessary. Depending on the crop, whether an annual crop or a multi-year root crop or tree bark crop, the product development timeline must take into consideration the transition period of the producer to organic production control and certification.

As an example, in the early 2000s, the authors contacted a European phytopharmaceutical extraction house to gauge their interest in producing a pharmacopoeial quality dry hydroalcoholic extract that would be both certified organic and kosher. The annual quantity was sufficient to consider co-development of a special extract, one that would not be offered to other customers. The extraction house was willing to have areas of their facility controlled for organic and kosher certified production of special extracts. The Procurement personnel at the extraction house were generally procuring conventional, not certified organic herbal material, for their standard extracts. Initially we suggested certain certified organic medicinal plant farms for them to qualify and add to their network of producers. Because the herbal material is produced as a 3-year root crop, the farmers require, annually, that all parties, the herbal tea brand, the extraction house, and the farm, agree on the quantity to plant each year (for harvest in 3 years). The brand must present its best guess of sales projections 3 years out in order to inform the farmer of what amount to plant under a guaranteed sale condition for harvest in 3 years. Furthermore, the extraction house does not have particle sizing, granulation, or density adjustment technologies. This means that we procure a powdered dry extract and ship it to a contract processor for granulation.

For an uninterrupted supply of this special extract, all stakeholders in the value chain, the farm(s), the extraction house, the contract granulator, and the herbal tea brand, must collaborate, plan years in advance, and each must maintain their certifications for supply chain visibility and evidence of sustainable production.

## Discussion and conclusion

While botanical extracts have become commonplace ingredients in botanical medicines, supplements and functional foods, their incorporation into medicinal tea formulas is a relatively new development (Block et al., 2016). The concept provides unique opportunities, such as the utilization of ingredients which are by nature not water-soluble and overcoming dosing constraints where the efficacious daily dose of a given ingredient cannot be reasonably administered in the tea format. In other words, extracts allow medicinal and wellness tea manufacturers to broaden their ingredient portfolio, tap into market segments or consumer needs that were hitherto inaccessible, improve consumer compliance, and are thus of great commercial interest.

At the same time, utilizing extracts in herbal tea formulas comes with challenges which have yet to be entirely overcome. Starting right at the source, botanical extracts that match the unique requirements for use in an infusion are to-date largely not available off-the-shelf, especially when pharmaceutical quality is required in conjunction with standards of sustainability (Chen et al., 2016). Extract development can be costly, time-consuming, and require close collaboration between the finished product and extract manufacturer, thus binding additional resources. Once such collaboration has been successfully established, unique physical parameters need to be observed for a botanical extract to be successfully integrated into an herbal tea formula, which include, among others, heat stability, solubility, dispersibility, and blend distribution—closely related to particle size and hardness, hygroscopicity, machinability, packaging and shelf-life *etc.*

By enabling the effective delivery of non-polar ingredients into a simple infusion, extracts (both native extracts and extracts that have undergone post-extraction processing) (Monagas et al., 2022) open many additional options for the formulation of effective medicinal infusions. While informed by traditional knowledge and insights on herbal preparation and delivery, the technologies described above offer new horizons in herbal pharmacy. However, there is room for improvement and refinement: first, additional progress can be made in creating final agglomerated extracts with desirable physical properties. Second, developing adequate packaging substrates with better oxygen and moisture barrier qualities that use sustainable materials will be necessary to help ensure that the sensory and medicinal qualities of the extracts and the formulations that include them are maintained throughout the shelf life of the final product.

The techniques explored above allow for a much wider range of ingredients to be considered in the formulation of teabag infusion products at an industrial scale. Due to the relative ease and convenience of the infusion process, this means that a wider range of consumers gain access to the benefits of herbal ingredients that might traditionally have been outside the scope of a teabag application (Poswal et al., 2019). Because extraction and post-extraction processing both liberate the ingredients’ relevant chemistry and assist in its solubility/dispersal into the final infusion as consumed, these herbs can participate in the formulation as true active ingredients.

Further challenges come with quality control and assurance of the finished product, in which the botanical extract needs to be identified and quantified among a potential matrix of other botanical ingredients that by far exceed the extract in weight and volume. While identification can generally be accomplished *via* thin-layer chromatographic methods, assays for the quantification of extracts in complex matrices are generally lacking due to the specificity required and need to be developed based on a formula-by-formula (matrix-by-matrix) basis (Reich and Frommenwiler, 2021).

In summary, while we have presented a number of successful approaches to address the specific requirements of utilizing botanical extracts in herbal tea formulas, our primary aim with this review is to create awareness among extract manufacturers, academia and standard-setting institutions of the specific needs in both manufacture and quality control for the herbal tea market.
